# Supplementary material for: HIVprotI: an integrated web based platform for prediction and design of HIV proteins inhibitors
Source: J Cheminform. 2018 Mar 9;10:12. doi: 10.1186/s13321-018-0266-y (PMC5845081; doi:10.1186/s13321-018-0266-y)
Supplement: Supplementary file 2 — Additional file 2. Source code of HIVProtI web server. [file 13321_2018_266_MOESM2_ESM.zip › HIVprotI_Source-code/hivprotI/weka-3-6-6/doc/weka/classifiers/trees/UserClassifier.html]

UserClassifier


---


|  |  |  |
| --- | --- | --- |
| **Overview** \| **Package** \| **Class** \| **Tree** \| **Deprecated** \| **Index** \| **Help** \| **Weka's home** | |  |
| **PREV CLASS**   NEXT CLASS | **FRAMES**    **NO FRAMES**     **All Classes** |  |
| SUMMARY: NESTED \| FIELD \| CONSTR \| METHOD | DETAIL: FIELD \| CONSTR \| METHOD |  |


---


## weka.classifiers.trees Class UserClassifier

```
java.lang.Object
  weka.classifiers.Classifier
      weka.classifiers.trees.UserClassifier
```

**All Implemented Interfaces:**: java.io.Serializable, java.lang.Cloneable, CapabilitiesHandler, Drawable, OptionHandler, RevisionHandler, TechnicalInformationHandler, TreeDisplayListener, VisualizePanelListener

---

``` public class UserClassifier extends Classifier implements Drawable, TreeDisplayListener, VisualizePanelListener, TechnicalInformationHandler ```

Interactively classify through visual means. You are Presented with a scatter graph of the data against two user selectable attributes, as well as a view of the decision tree. You can create binary splits by creating polygons around data plotted on the scatter graph, as well as by allowing another classifier to take over at points in the decision tree should you see fit.
